# Describing the population experiencing COVID-19 vaccine breakthrough following second vaccination in England: a cohort study from OpenSAFELY

**DOI:** 10.1186/s12916-022-02422-0

**Published:** 2022-07-05

**Authors:** The OpenSAFELY Collaborative, Amelia Green, Helen Curtis, William Hulme, Elizabeth Williamson, Helen McDonald, Krishnan Bhaskaran, Christopher Rentsch, Anna Schultze, Brian MacKenna, Viyaasan Mahalingasivam, Laurie Tomlinson, Alex Walker, Louis Fisher, Jon Massey, Colm Andrews, Lisa Hopcroft, Caroline Morton, Richard Croker, Jessica Morley, Amir Mehrkar, Seb Bacon, David Evans, Peter Inglesby, George Hickman, Tom Ward, Simon Davy, Rohini Mathur, John Tazare, Rosalind Eggo, Kevin Wing, Angel Wong, Harriet Forbes, Chris Bates, Jonathan Cockburn, John Parry, Frank Hester, Sam Harper, Ian Douglas, Stephen Evans, Liam Smeeth, Ben Goldacre

**Affiliations:** 1 Bennett Institute for Applied Data Science, Nuffield Department of Primary Care Health Sciences, University of Oxford, Oxford, OX2 6GG UK; 2 London School of Hygiene and Tropical Medicine, Keppel Street, London, WC1E 7HT UK; 3 TPP, TPP House, 129 Low Lane, Horsforth, Leeds, LS18 5PX UK

**Keywords:** COVID-19, Vaccine breakthrough, EHR data

## Abstract

**Background:**

While the vaccines against COVID-19 are highly effective, COVID-19 vaccine breakthrough is possible despite being fully vaccinated. With SARS-CoV-2 variants still circulating, describing the characteristics of individuals who have experienced COVID-19 vaccine breakthroughs could be hugely important in helping to determine who may be at greatest risk.

**Methods:**

With the approval of NHS England, we conducted a retrospective cohort study using routine clinical data from the OpenSAFELY-TPP database of fully vaccinated individuals, linked to secondary care and death registry data and described the characteristics of those experiencing COVID-19 vaccine breakthroughs.

**Results:**

As of 1st November 2021, a total of 15,501,550 individuals were identified as being fully vaccinated against COVID-19, with a median follow-up time of 149 days (IQR: ​107–179). From within this population, a total of 579,780 (<4%) individuals reported a positive SARS-CoV-2 test. For every 1000 years of patient follow-up time, the corresponding incidence rate (IR) was 98.06 (95% CI 97.93–98.19). There were 28,580 COVID-19-related hospital admissions, 1980 COVID-19-related critical care admissions and 6435 COVID-19-related deaths; corresponding IRs 4.77 (95% CI 4.74–4.80), 0.33 (95% CI 0.32–0.34) and 1.07 (95% CI 1.06–1.09), respectively. The highest rates of breakthrough COVID-19 were seen in those in care homes and in patients with chronic kidney disease, dialysis, transplant, haematological malignancy or who were immunocompromised.

**Conclusions:**

While the majority of COVID-19 vaccine breakthrough cases in England were mild, some differences in rates of breakthrough cases have been identified in several clinical groups. While it is important to note that these findings are simply descriptive and cannot be used to answer why certain groups have higher rates of COVID-19 breakthrough than others, the emergence of the Omicron variant of COVID-19 coupled with the number of positive SARS-CoV-2 tests still occurring is concerning and as numbers of fully vaccinated (and boosted) individuals increases and as follow-up time lengthens, so too will the number of COVID-19 breakthrough cases. Additional analyses, to assess vaccine waning and rates of breakthrough COVID-19 between different variants, aimed at identifying individuals at higher risk, are needed.

**Supplementary Information:**

The online version contains supplementary material available at 10.1186/s12916-022-02422-0.

## Background

The vaccination programme for COVID-19 in the United Kingdom (UK) was started on 8 December 2020. Vaccination was in order of priority groups determined by the Joint Committee on Vaccination and Immunisation (JCVI) expert advisory group [1]: initially to people aged 80+ years, health and care workers and care home residents, people aged 70–79 and those extremely clinically vulnerable, followed by remaining adults in order of decreasing age or at increased risk. Three COVID-19 vaccines, approved by the Medicines and Healthcare products Regulatory Agency (MHRA) and requiring two doses to produce maximum protection, are currently in use in the United Kingdom (UK) [2]. To allow a higher percentage of the population to receive one vaccine dose quicker, second doses initially followed a 12-week interval from the first. This interval has since been shortened. These vaccines and their date of first administration and current second dose schedule are as follows: the Pfizer-BioNTech BNT162b2 mRNA COVID-19 vaccine (BNT162b2; first administered 8 Dec 2020, second dose at least 21 days later); the Oxford-AstraZeneca ChAdOx1 nCoV-19 vaccine (ChAdOx1; first administered 4 Jan 2021, second dose between 4 and 12 weeks later); and the Moderna mRNA-1273 vaccine (mRNA-1273; first administered 7 April 2021, second dose 28 days later). As of November 1st, 2021 78.7% of individuals aged over 16 years in England had been fully vaccinated (i.e., ≥14 days have passed since the receipt of their second dose of a COVID-19 vaccine) [3].

The vaccines against COVID-19 are considered to be highly effective and there is strong evidence that the UK’s COVID-19 vaccination programme has reduced infection and severe outcomes in vaccinated individuals [4–6]. However, breakthrough infections after vaccination against SARS-CoV-2 are increasingly reported [7] and there are concerns that the effectiveness of the vaccines may fade over time, due to the vaccines being less effective against the delta variant, and/or the waning of the immune response [8]. In addition, as no vaccine is 100% effective, COVID-19 vaccine breakthrough is likely (i.e., a small number of people will still get sick, be hospitalised, or die from COVID-19, despite being fully vaccinated). Describing individuals who have experienced a COVID-19 vaccine breakthrough could provide the first indication that the COVID-19 vaccine is less effective in certain groups and could be hugely important in helping to determine who may be at greatest risk and therefore might benefit most from booster doses.

To that end we used the new secure data analytics platform, OpenSAFELY [9] (built by our group on behalf of NHS England to support analysis of important questions related to COVID-19) to describe breakthrough COVID-19 amongst fully vaccinated individuals in England; and to describe how breakthrough COVID-19 varied between priority groups and by clinical and demographic characteristics.

## Methods

### Data sources

This OpenSAFELY study was conducted using the OpenSAFELY-TPP database which contains records for approximately 24 million people currently registered with GP surgeries using TPP SystmOne software (approximately 40% of the English population).

### Study population

The base population consisted of all individuals aged 16 years or over with records indicating that they had received two COVID-19 vaccination doses since 8 December 2020 (the earliest vaccination date in England) and who were still alive and registered 2 weeks after their second dose. Individuals were excluded if they tested positive for SARS-CoV-2 or had been hospitalised or died due to COVID-19 within the 2 weeks after their second dose. Follow-up started 14 days after an individual’s second dose of the COVID-19 vaccination (the point by which individuals were classified as being fully vaccinated) and individuals were followed up until 1st November 2021 or until the first occurrence of the outcome of interest, death, and practice de-registration.

### Outcomes

Four outcomes were assessed: positive SARS-CoV-2 swab test, via SGSS and based on swab date (we did not distinguish between symptomatic and asymptomatic infection for this outcome, nor PCR and lateral flow testing); COVID-19-related hospital admissions via Hospital Episode Statistics (HES) in-patient records (using both primary and non-primary diagnosis codes); COVID-19-related critical care admissions via HES and COVID-19-related death, via linked death registry records, which included individuals who died within 28 days of positive SARS-CoV-2 test or who had COVID-19 mentioned on the death certificate as one of the causes. COVID-19 hospitalisation variables were derived using two ICD-10 COVID-19 diagnosis codes; U071, U072 [10]. Outcomes were only included if they occurred 14 days or more after an individual's second dose of a COVID-19 vaccination.

### Priority groups for vaccination

Individuals were classified into seven priority groups (Table 1), based on the Joint Committee on Vaccination and Immunisation (JCVI) priority groups [11] using SNOMED-CT codelists and logic defined in the national COVID-19 Vaccination Uptake Reporting Specification developed by PRIMIS [12]. Individuals were assigned only to their highest priority group and not included again as part of any other priority group. In line with the national reporting specification, most criteria were ascertained using the latest available data at the time of analysis, with the exception of age which was calculated as at 31 March 2021 as recommended by Public Health England.

**Table 1:** Priority groups for vaccination

JCVI risk group	Priority group	
	Description	Name
1	Residents in a care home for older adults, aged 65+	Care home
2	All those 80 years of age and over	80+
	Health and social care workers	Health/care workers
3	All those 70 years of age and over	70–79
4	Clinically extremely vulnerable individuals	CEV
5, 7, 8, 9	All those 50 years of age and over	50-69
6	“At Risk” individuals, some misclassified health/care workers and care home residents (except those age 70+ or shielding who will be included in another group), carers, household contacts of those at increased risk, and those not in priority groups who had the vaccine opportunistically, e.g. by responding to calls to use up excess doses.	Others not in the above groups

### Key demographic and clinical characteristics

Key clinical and demographic groups which were considered to have a higher possibility of experiencing a COVID-19 vaccine breakthrough were identified from previous studies [13–17]. This included age, sex, body mass index (BMI; kg/m^2^), smoking status, deprivation (measured by the index of multiple deprivation (IMD) as quintiles, ethnicity, NHS region of patient’s general practice, asplenia, asthma, blood pressure, cancer, chronic kidney disease, diabetes mellitus, dialysis, heart disease, haematological malignancy, immunocompromised, learning disability, liver disease, neurological diseases, respiratory disease, severe mental illness and transplant. Other variables considered were time since being fully vaccinated, time between vaccinations and any evidence of a prior SARS-CoV-2 infection.

### Codelists and implementationg

Information on all variables were obtained from primary care records by searching TPP SystmOne records for specific coded data. Detailed information on compilation and sources for every individual codelist is available at https://codelists.opensafely.org/ and the lists are available for inspection and re-use by the broader research community.

### Missing data

Individuals with missing ethnicity, IMD and region were included as “Unknown”.

### Statistical methods

Simple descriptive statistics were used to summarise the counts and rates of COVID-19 vaccine breakthrough. Rates for each outcome were estimated by dividing the count by person-years, with 95% confidence intervals. Counts and rates were stratified by initial priority groups for all outcomes and by selected clinical and demographic groups.

### Software and reproducibility

Data management and analysis was performed using the OpenSAFELY software libraries, Python 3 and R version 4.0.2. All code for the OpenSAFELY platform for data management, analysis and secure code execution is shared for review and re-use under open licenses at GitHub.com/OpenSAFELY. All code for data management and analysis for this paper is shared for scientific review and re-use under open licenses on GitHub (https://github.com/opensafely/covid-19-vaccine-breakthrough).

### Patient and public involvement

Any patient or member of the public is invited to contact us at https://opensafely.org/ regarding this study or the broader OpenSAFELY project.

## Results

### Study population

**Fig. 1:**
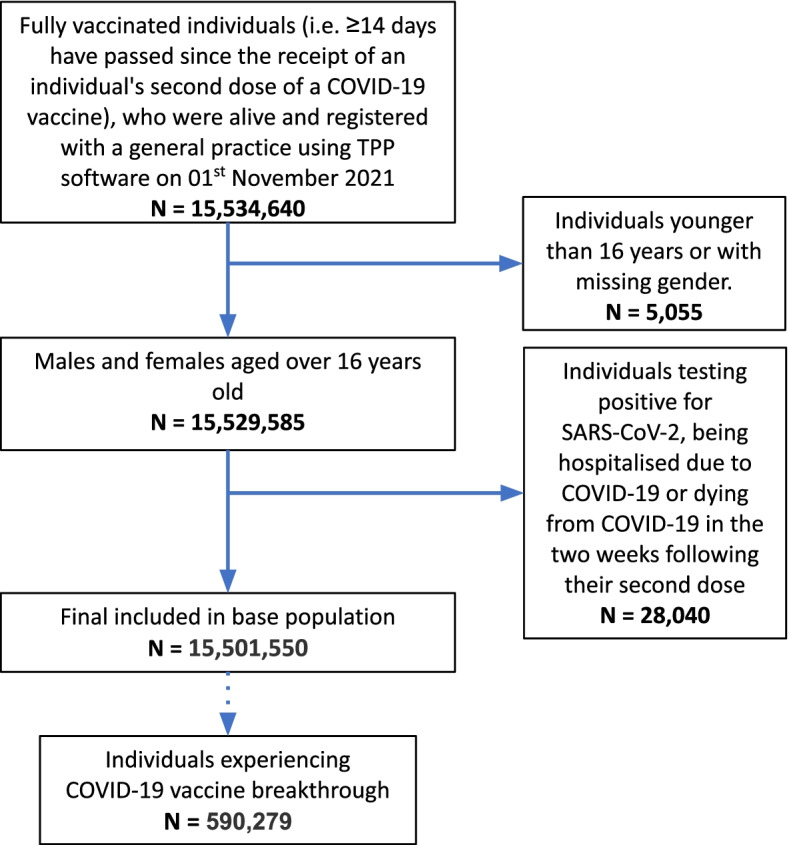
Inclusion/exclusion flowchart

Out of approximately 24 million patients, 15,501,550 individuals were identified as being fully vaccinated against COVID-19 and included in this study (Fig. 1). The median follow-up time was 149 days (interquartile range: 107–179 days). Since being fully vaccinated, 8,370,837 (54%) of the base population had at least one record for a (positive/negative) SARS-CoV-2 test recorded, with a positivity rate of 3.17%. Testing behaviours varied between priority groups with individuals in care homes testing more regularly than other groups; 91% of care home residents had 3+ SARS-CoV-2 tests since being fully vaccinated vs 41–73% in other groups. The total number of COVID-19 vaccine breakthrough cases was 590,279 (<4%). Table 2 shows the number and rate of individuals fully vaccinated broken down by initial JCVI priority groups, along with the number and rate (per 1000 patient years) of each outcome.

**Table 2:** Number of individuals fully vaccinated (2 doses + 2 weeks) in initial priority groups in OpenSAFELY-TPP, and number and rate with each outcome

Priority group	Fully vaccinated						Positive SARS-CoV-2 test^a^			COVID-19-related hospital admission^a^		COVID-19-related critical care admissiona^a^		COVID-19-related death^a^	
	Count^b^	Follow-up time, medium days (IQR)	Tests conducted (%)^c^				Positivity (%)	Events^b^ (PYs)	Incidence rate per 1000 person-years (95% CI)	Events^b^ (PYs)	Incidence rate per 1000 person-years (95% CI)	Events^b^ (PYs)	Incidence rate per 1000 person-years (95% CI)	Events^b^ (PYs)	Incidence rate per 1000 person-years (95% CI)
			0	1	2	3+									
**All**	15,501,550	149 (107–179)	46	19	10	19	579,780 (5,912,498)	3.17	98.06 (97.93–98.19)	28,580 (,5993,535)	4.77 (4.74–4.8)	1980 (5996007)	0.33 (0.32–0.34)	6435 (5,996,178)	1.07 (1.06–1.09)
**Care home** (priority group 1)	98,400	200 (180–207)	9	5	4	82	3110 (48,207)	0.90	64.51 (63.37–65.68)	755 (48,600)	15.58 (15.02–16.15)	REDACTED	REDACTED	650 (48,674)	13.37 (12.86–13.91)
**80+** (priority group 2)	1,073,145	200 (191–213)	59	14	7	20	15,945 (597,368)	1.96	26.69 (26.48–26.9)	7545 (598,868)	12.60 (12.45–12.74)	180 (599,613)	0.30 (0.28–0.32)	2785 (599,626)	4.64 (4.56–4.73)
**Health/care workers** (priority groups 1–2)	555,820	200 (182–211)	27	21	14	39	30,045 (282,813)	3.31	106.23 (105.62–106.84)	440 (287,822)	1.53 (1.46–1.60)	30 (287,862)	0.10 (0.08–0.12)	20 (287,866)	0.07 (0.05–0.08)
**70–79** (priority groups 3–4)	1,990,755	184 (176–191)	56	17	8	19	41,325 (985,544)	2.16	41.93 (41.73–42.14)	6875 (990,497)	6.94 (6.86–7.03)	525 (991,111)	0.53 (0.51–0.56)	1640 (991,147)	1.66 (1.62–1.70)
**CEV (age 16–69)** (priority group 4)	725,045	171 (157–181)	42	18	10	30	34,820 (322,885)	2.92	107.84 (107.27–108.42)	4560 (327,932)	13.90 (13.7–14.11)	565 (328,308)	1.73 (1.66–1.80)	785 (328,361)	2.39 (2.31–2.48)
**50–69** (priority groups 5–9)	4,730,155	151 (138–166)	47	18	9	26	184,890 (1,946,451)	2.27	94.99 (94.77–95.21)	5075 (1,973,346)	2.57 (2.54–2.61)	515 (1,973,743)	0.26 (0.25–0.27)	495 (1,973,791)	0.25 (0.24–0.26)
Others not in the above groups^d^	15,501,550	149 (107–179)	46	19	10	19	579,780 (5,912,498)	3.17	98.06 (97.93–98.19)	28,580 (5,993,535)	4.77 (4.74–4.80)	1980 (5,996,007)	0.33 (0.32–0.34)	6435 (5,996,178)	1.07 (1.06–1.09)

*CEV* clinically extremely vulnerable.  ^a^All outcomes calculated as of 01st November 2021. ^b^All counts (of people and events) have been rounded to the nearest 5 and counts. ^c^Tests conducted is a count of (positive and negative) test results and may include multiple tests for an individual person. ^d^Others includes: others in priority groups (“At Risk”), some misclassified health/care workers and care home residents (except those age 70+ or shielding who will be included in another group), carers, household contacts of those at increased risk, and those not in priority groups who had the vaccine opportunistically, e.g., by responding to calls to use up excess doses

### Positive SARS-CoV-2 test

**Fig. 2:**
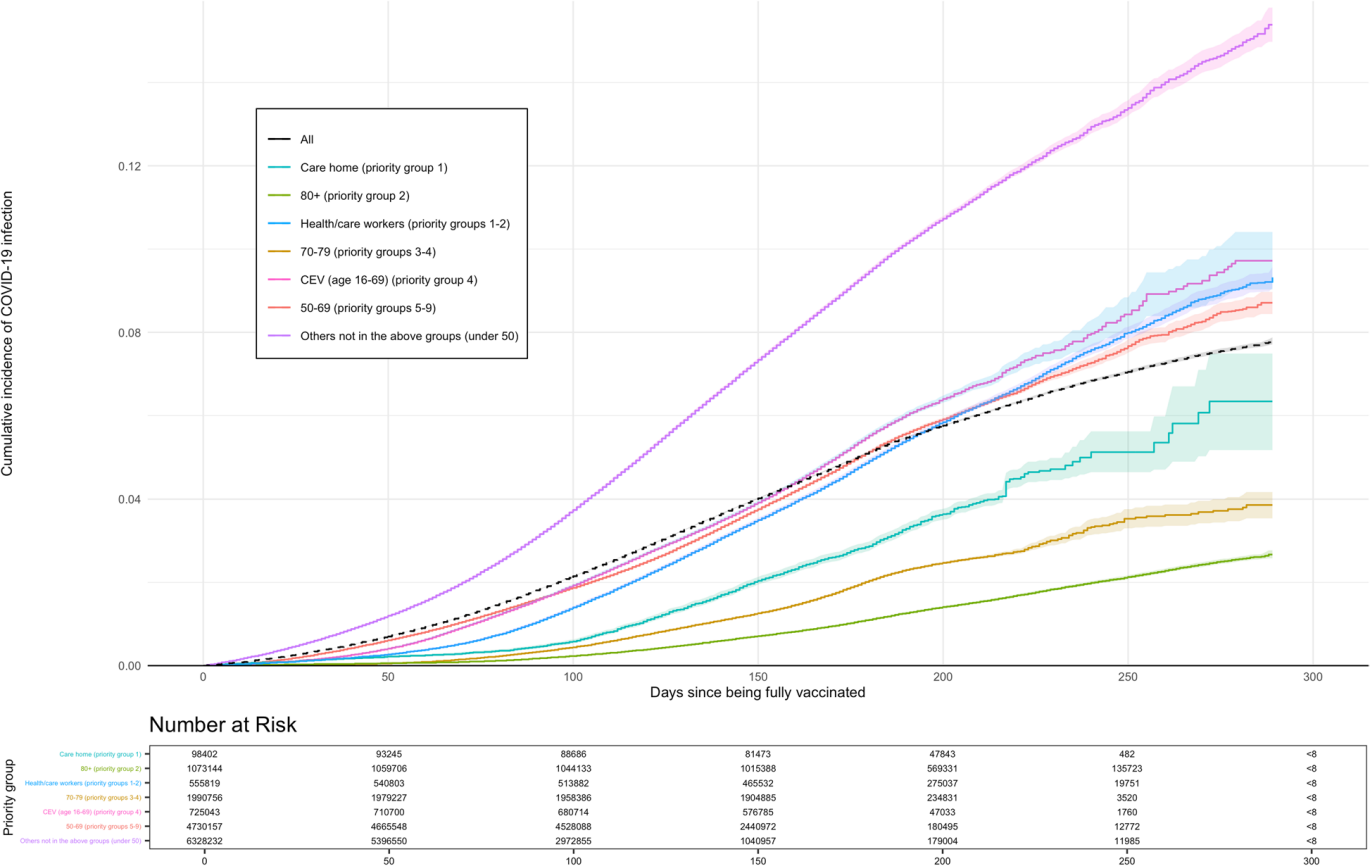
Kaplan-Meir plot for positive SARS-CoV-2 test over time, by priority group

In fully vaccinated individuals, the median number of days to a positive test for SARS-CoV-2 was 99 (IQR 63–134 days) with a total of 579,780 individuals testing positive for SARS-CoV-2 (about 1 in 25 or <4%). For every 1000 years of patient follow-up time, the corresponding incidence rate was ​98.06 (95% CI 97.93–98.19). Within the initial JCVI priority groups, positive SARS-CoV-2 test rates were highest in the CEV group (107.84, 95% CI 107.27-108.42) and lowest in those over 80 years of age (26.69, 95% CI 26.48-26.90). The overall cumulative incidence of positive SARS-CoV-2 tests at 275 days from the study start date was <0.1% (Fig. 2).

When broken down into clinical/demographic groups and comorbidities (Table 3), rates of individuals testing positive for SARS-CoV-2 were highest in 40–40 years old (179.96, 95% CI 179.50–180.42) and lowest in individuals over 80 years of age (28.90, 95% CI 28.69–29.11). Rates were higher in females than in males: 102.60 (95% CI 102.42–102.78) vs 92.76 ( 95% CI 92.58–92.95), respectively. Comorbidities with the highest rates of positive SARS-CoV-2 tests included kidney transplant, dialysis and immunocompromised: 143.51 (95% CI 136.46–150.93), 134.75 (95% CI 124.32–146.06), and 91.53 (95% CI 90.91–92.15), respectively. Rates of positive SARS-CoV-2 tests were lowest 4–8 weeks after being fully vaccinated and highest at 12+ weeks: 48.65 (95% CI 47.97–49.33) vs 100.3 (95% CI 100.16–100.43), respectively.

**Table 3:** Count and rates of breakthrough positive SARS-CoV-2 tests and hospitalisation and death in fully vaccinated individuals in OpenSAFELY-TPP, broken down by selected clinical and demographic groups

Clinical/demographic group	Category	Fully vaccinated						Positive SARS-CoV-2 test^a^			Hospitalised with COVID-19^a^		COVID-19-related critical care admission^a^		COVID-19 related death^a^	
		Count^ab^	Follow-up time, medium days (IQR)	Tests conducted (%)^c^				Positivity (%)	Events^ab^ (PYs)	Incidence rate per 1000 person-years (95% CI)	Events^ab^ (PYs)	Incidence rate per 1000 person-years (95% CI)	Events^ab^ (PYs)	Incidence rate per 1000 person-years (95% CI)	Events^ab^ (PYs)	Incidence rate per 1000 person-years (95% CI)
				0	1	2	3+									
**Age**	16–29	2,227,120	66 (47–129)	45	21	11	22	63,620 (516,289)	5.47	123.22 (122.74–123.71)	1000 (527,073)	1.90 (1.84–1.96)	40 (527,145)	0.07 (0.06–0.08)	10 (527,150)	0.02 (0.01–0.02)
	30–39	2,289,165	94 (72–145)	42	21	12	25	93,375 (660,313)	4.50	141.41 (140.95–141.88)	1550 (673,014)	2.30 (2.25–2.36)	80 (673,118)	0.12 (0.10–0.13)	35 (673,127)	0.05 (0.04–0.06)
	40–50	2,459,075	126 (103–155)	40	20	11	29	153,210 (851,368)	3.86	179.96 (179.5–180.42)	2320 (871,625)	2.66 (2.61–2.72)	210 (871,799)	0.24 (0.22–0.26)	120 (871,821)	0.14 (0.12–0.15)
	50–59	2,942,440	146 (135–163)	43	19	10	28	136,470 (1,188,958)	2.49	114.78 (114.47–115.09)	3850 (1,209,351)	3.18 (3.13–3.23)	420 (1,209,663)	0.35 (0.33–0.37)	370 (1,209,705)	0.31 (0.29–0.32)
	60–69	2,421,245	163 (150–174)	49	18	9	24	72,735 (1,064,063)	1.94	68.36 (68.1–68.61)	4715 (1,074,117)	4.39 (4.33–4.45)	530 (1,074,498)	0.49 (0.47–0.52)	845 (1,074,542)	0.78 (0.76–0.81)
	70–79	2,016,365	184 (176–191)	56	17	8	19	42,075 (998433)	2.11	42.14 (41.93–42.34)	7050 (1,003,480)	7.03 (6.94–7.11)	530 (1,004,114)	0.53 (0.51–0.55)	1720 (1,004,149)	1.71 (1.67–1.76)
	80 +	1,146,150	200 (191–213)	55	14	7	24	18,295 (633,074)	1.72	28.9 (28.69–29.11)	8095 (634,874)	12.75 (12.61–12.89)	180 (635670)	0.29 (0.27–0.31)	3345 (635,683)	5.26 (5.17–5.35)
**Sex**	Female	8,072,115	155 (116–184)	43	19	10	28	326,565 (3,182,819)	2.69	102.6 (102.42–102.78)	13,500 (3,228,984)	4.18 (4.14–4.22)	740 (3,230,164)	0.23 (0.22–0.24)	2655 (3,230,233)	0.82 (0.81–0.84)
	Male	7,429,435	144 (99–174)	50	19	10	21	253,215 (2,729,679)	4.04	92.76 (92.58–92.95)	15,080 (2,764,551)	5.46 (5.41–5.50)	1250 (2,765,843)	0.45 (0.44–0.46)	3780 (2,765,945)	1.37 (1.34–1.39)
**BMI (kg/m**^**2**^**)**	Not obese	12,078,955	145 (98–178)	46	19	10	25	429,315 (4,498,201)	3.20	95.44 (95.3–95.59)	18,330 (4,558,086)	4.02 (3.99–4.05)	1010 (4,559,694)	0.22 (0.21–0.23)	4475 (4,559,783)	0.98 (0.97–1.00)
	Obese (>30)	3,422,600	159 (135–181)	46	19	10	26	150,465 (1,414,297)	3.07	106.39 (106.11–106.66)	10,255 (1,435,449)	7.14 (7.07–7.21)	980 (1,436,313)	0.68 (0.66–0.71)	1965 (1,436,395)	1.37 (1.34–1.40)
**Smoking**	Non-smoker and unknown	7,833,690	143 (91–176)	45	19	10	26	291,000 (2,835,776)	3.19	102.62 (102.43–102.81)	9960 (2,876,640)	3.46 (3.43–3.50)	675 (2,877,506)	0.24 (0.23–0.24)	1860 (2,877,571)	0.65 (0.63–0.66)
	Current and former	7,667,865	156 (125–182)	47	19	10	24	288,780 (3,076,722)	3.14	93.86 (93.68–94.03)	18,620 (3,116,894)	5.97 (5.93–6.02)	1310 (3,118,501)	0.42 (0.41–0.43)	4575 (3,118,607)	1.47 (1.45–1.49)
**Ethnicity**	White	11,895,195	154 (119–182)	44	19	10	26	466,170 (4,688,366)	3.08	99.43 (99.29–99.58)	25,500 (4,753,382)	5.36 (5.33–5.40)	1760 (4,755,592)	0.37 (0.36–0.38)	5905 (4,755,742)	1.24 (1.23–1.26)
	Mixed	151,275	129 (76–164)	44	20	11	25	4980 (50,030)	3.78	99.56 (98.16–100.98)	195 (50,764)	3.82 (3.56–4.11)	20 (50,779)	0.35 (0.28–0.45)	25 (50,780)	0.51 (0.42–0.62)
	Asian or Asian British	901,520	134 (81–170)	55	19	10	17	29,830 (309,199)	4.24	96.48 (95.92–97.04)	1735 (313,404)	5.54 (5.40–5.67)	120 (313,565)	0.39 (0.36–0.43)	300 (313,577)	0.95 (0.90–1.01)
	Black or Black British	224,740	138 (87–172)	49	18	9	23	5930 (78,696)	2.98	75.37 (74.39–76.35)	415 (79,581)	5.20 (4.95–5.46)	25 (79,614)	0.34 (0.28–0.41)	50 (79,616)	0.63 (0.55–0.72)
	Other ethnic groups	242,335	122 (79–159)	50	19	10	20	5970 (78,690)	3.66	75.85 (74.88–76.84)	295 (79,509)	3.69 (3.48–3.91)	30 (79,531)	0.38 (0.31–0.45)	55 (79,534)	0.67 (0.58–0.76)
	Unknown	2,086,480	135 (82–164)	53	18	9	21	66,900 (707,517)	3.36	94.56 (94.19–94.92)	445 (716,894)	0.62 (0.59–0.65)	30 (716,926)	0.04 (0.03–0.05)	105 (716,928)	0.14 (0.13–0.16)
**IMD quintile**	1 (most deprived)	2,519,265	146 (100–176)	54	18	9	20	96,800 (927,354)	3.56	104.39 (104.05–104.72)	6890 (941,680)	7.32 (7.23–7.41)	460 (942,314)	0.49 (0.46–0.51)	1535 (942,355)	1.63 (1.59–1.67)
	2	2,871,570	148 (101–178)	49	19	9	23	105,430 (1,074,696)	3.30	98.10 (97.80–98.4)	5860 (1,089,699)	5.38 (5.31–5.45)	410 (1,090,201)	0.38 (0.36–0.39)	1310 (1,090,236)	1.20 (1.17–1.23)
	3	3,318,680	150 (109–180)	46	19	10	25	119,680 (1,274,137)	3.06	93.93 (93.66–94.2)	5595 (1,290,631)	4.33 (4.28–4.39)	390 (1,291,108)	0.30 (0.29–0.32)	1265 (1,291,143)	0.98 (0.95–1.01)
	4	3,274,460	151 (113–181)	44	19	10	27	121,740 (1,272,731)	3.07	95.65 (95.38–95.93)	5210 (1,289,336)	4.04 (3.98–4.10)	380 (1,289,775)	0.29 (0.28–0.31)	1195 (1,289,806)	0.92 (0.90–0.95)
	5 (least deprived)	3,115,025	152 (115–181)	41	19	11	29	120,245 (1,220,876)	2.98	98.49 (98.21–98.77)	4395 (1,237,218)	3.55 (3.50–3.61)	310 (1,237,580)	0.25 (0.24–0.26)	1020 (1,237,606)	0.82 (0.80–0.85)
	Unknown	402,555	139 (87–173)	43	20	11	26	15,885 (142,704)	3.64	111.31 (110.43–112.19)	635 (144,971)	4.37 (4.20–4.55)	45 (145,029)	0.30 (0.25–0.35)	120 (145,032)	0.82 (0.75–0.90)
**Region**	London	891,660	129 (83–170)	40	20	12	28	21,970 (304,836)	4.19	72.07 (71.59–72.56)	1100 (308,097)	3.57 (3.46–3.68)	70 (308,184)	0.23 (0.21–0.26)	220 (308,190)	0.72 (0.67–0.77)
	East of England	3,629,745	149 (104–179)	44	19	10	27	113,725 (1,380,061)	2.82	82.41 (82.16–82.65)	5105 (1,394,975)	3.66 (3.61–3.71)	410 (1,395,387)	0.29 (0.28–0.31)	1215 (1,395,416)	0.87 (0.84–0.89)
**Region** (continued)	East Midlands	2,702,030	150 (113–177)	48	19	10	24	108,255 (1,029,678)	3.47	105.14 (104.82–105.46)	5480 (1,044,633)	5.24 (5.17–5.32)	420 (1,045,072)	0.4 (0.38–0.42)	1215 (1,045,111)	1.16 (1.13–1.20)
	North East	745,775	150 (115–181)	49	20	10	22	37,590 (289,494)	4.54	129.85 (129.18–130.52)	2170 (294,874)	7.36 (7.21–7.52)	140 (295,080)	0.47 (0.43–0.51)	495 (295,093)	1.68 (1.61–1.76)
	North West	1,402,645	150 (115–180)	49	19	9	23	63,570 (541,871)	3.55	117.32 (116.85–117.78)	3445 (551,601)	6.24 (6.14–6.35)	265 (551,937)	0.48 (0.45–0.51)	780 (551,962)	1.42 (1.37–1.47)
	South East	1,078,015	151 (108–180)	43	19	10	27	34,655 (417,431)	2.65	83.02 (82.58–83.47)	1715 (422,133)	4.06 (3.96–4.16)	110 (422,262)	0.27 (0.24–0.29)	370 (422,271)	0.88 (0.84–0.93)
	South West	2,314,235	152 (111–180)	45	19	10	27	83,030 (896,729)	2.56	92.59 (92.27–92.91)	3460 (907,535)	3.81 (3.75–3.88)	185 (907,805)	0.20 (0.19–0.22)	760 (907,822)	0.83 (0.81–0.87)
	West Midlands	548,600	149 (109–179)	52	18	9	22	21,340 (208,833)	3.69	102.19 (101.49–102.89)	1425 (212,055)	6.73 (6.55–6.91)	105 (212,201)	0.50 (0.45–0.55)	300 (212,211)	1.40 (1.33–1.49)
	Yorkshire and The Humber	2,179,520	151 (111–181)	50	19	9	22	95,360 (840,635)	3.44	113.44 (113.07–113.81)	4670 (854,660)	5.46 (5.38–5.54)	280 (855,109)	0.33 (0.31–0.35)	1080 (855,132)	1.26 (1.22–1.30)
	Unknown	9325	116 (67–164)	44	20	11	25	280 (2929)	3.85	96.27 (90.7–102.17)	15 (2969)	4.71 (3.61–6.16)	REDACTED	REDACTED	REDACTED	REDACTED
**Asplenia**		108,350	170 (154–186)	40	19	11	30	4890 (48,994)	2.62	99.79 (98.37–101.22)	280 (49,718)	5.59 (5.27–5.94)	15 (49744)	0.32 (0.25–0.41)	55 (49,746)	1.13 (0.98–1.29)
**Asthma**		2,336,650	151 (110–178)	42	20	11	27	105,015 (893,242)	3.43	117.57 (117.21–117.93)	5900 (908,166)	6.5 (6.41–6.58)	425 (908,684)	0.47 (0.44–0.49)	1070 (908,724)	1.18 (1.14–1.21)
**Blood pressure**	Normal	3,190,995	137 (87–171)	41	20	11	28	136,085 (1,113,098)	3.44	122.26 (121.93–122.59)	4975 (1,132,373)	4.4 (4.33–4.46)	260 (1,132,792)	0.23 (0.22–0.24)	1185 (1,132,816)	1.05 (1.02–1.08)
	Elevated/high	3,428,615	151 (122–179)	46	19	10	25	136,200 (1,347,493)	3.08	101.08 (100.8–101.35)	6565 (1,366,494)	4.8 (4.74–4.86)	480 (1,367,064)	0.35 (0.34–0.37)	1420 (1,367,106)	1.04 (1.01–1.07)
	Unknown	8,881,945	152 (113–181)	48	19	10	24	307,500 (3,451,907)	3.09	89.08 (88.92–89.24)	17,040 (3,494,668)	4.88 (4.84–4.91)	1250 (3,496,150)	0.36 (0.35–0.37)	3830 (3,496,255)	1.10 (1.08–1.11)
**Cancer (non-haematological)**		891,935	180 (160–194)	45	17	9	28	25,625 (426,015)	2.08	60.15 (59.77–60.52)	4065 (429,286)	9.47 (9.32–9.62)	235 (429,659)	0.54 (0.51–0.58)	1305 (429,678)	3.04 (2.96–3.13)
**Chronic kidney disease**	Stage 3a	529,000	187 (173–201)	53	16	8	23	12,170 (268,808)	2.18	45.27 (44.87–45.69)	3370 (270,184)	12.48 (12.27–12.7)	190 (270,500)	0.70 (0.65–0.75)	1080 (270,514)	4.00 (3.88–4.12)
	Stage 3b	206,450	192 (178–205)	52	14	7	27	4450 (107,113)	2.16	41.56 (40.95–42.19)	2105 (107,527)	19.59 (19.16–20.02)	95 (107,716)	0.89 (0.80–0.99)	795 (107,722)	7.39 (7.13–7.66)
	Stage 4	51,210	191 (174–204)	47	13	8	33	1280 (25,907)	2.19	49.41 (48.05–50.81)	780 (26,000)	29.92 (28.87–31.01)	45 (26,064)	1.80 (1.56–2.09)	335 (26067)	12.85 (12.17–13.57)
	Stage 5	5505	184 (164–200)	29	12	7	52	215 (2607)	1.75	82.47 (77.04–88.29)	130 (2627)	49.49 (45.33–54.02)	15 (2638)	5.31 (4.06–6.93)	45 (2638)	17.81 (15.4–20.61)
**Diabetes**		1,476,880	173 (157–191)	50	17	9	24	52,540 (683,694)	2.80	76.85 (76.51–77.18)	8645 (690,614)	12.52 (12.39–12.65)	740 (691,375)	1.07 (1.03–1.11)	2255 (691,436)	3.26 (3.19–3.33)
**Dialysis**	Previous kidney transplant	2345	183 (172–198)	25	15	9	51	155 (1143)	2.54	134.75 (124.32–146.06)	80 (1159)	70.73 (63.34–78.99)	25 (1165)	19.74 (16.03–24.32)	30 (1167)	25.71 (21.42–30.86)
	No previous kidney transplant	10,505	180 (165–199)	22	9	7	63	535 (4982)	1.38	107.19 (102.65–111.93)	230 (5048)	45.36 (42.46–48.46)	25 (5068)	5.33 (4.40–6.46)	65 (5070)	13.22 (11.7–14.93)
**Heart disease**		1,745,845	180 (163–194)	48	17	9	26	51,745 (844,423)	2.38	61.28 (61.01–61.55)	10,590 (850,928)	12.45 (12.33–12.57)	650 (851,904)	0.76 (0.73–0.79)	3405 (851,951)	4.00 (3.93–4.07)
**Haematological malignancy**		103,625	180 (166–193)	42	17	9	31	3725 (49,945)	2.91	74.56 (73.35–75.79)	1225 (50,386)	24.35 (23.67–25.06)	140 (50,481)	2.79 (2.57–3.04)	435 (50,492)	8.62 (8.21–9.04)
**Immunocompromised**		508,930	177 (159–191)	42	18	10	30	21,695 (237,016)	2.90	91.53 (90.91–92.15)	3950 (240,002)	16.45 (16.19–16.71)	450 (240,303)	1.88 (1.79–1.97)	1220 (240,339)	5.08 (4.94–5.23)
**Learning disability**		102,730	160 (146–177)	47	13	6	33	3230 (43,892)	1.78	73.57 (72.28–74.87)	315 (44,339)	7.06 (6.67–7.47)	30 (44,367)	0.65 (0.54–0.79)	50 (44371)	1.13 (0.98–1.30)
**Liver disease**		348,855	164 (144–182)	45	19	10	26	14,020 (151,587)	2.96	92.5 (91.72–93.29)	1620 (153,529)	10.55 (10.29–10.81)	130 (153,667)	0.85 (0.78–0.93)	360 (153,678)	2.36 (2.23–2.48)
**Neurological disease**		874,635	176 (156–193)	45	16	8	30	28,410 (407,866)	2.29	69.66 (69.24–70.07)	5075 (411,606)	12.33 (12.16–12.51)	245 (412,073)	0.59 (0.55–0.63)	1685 (412,095)	4.09 (3.99–4.19)
**Respiratory disease**		581,930	180 (166–193)	50	17	9	25	16,785 (282,315)	2.53	59.45 (58.99–59.91)	4900 (284,321)	17.24 (16.99–17.49)	305 (284,751)	1.07 (1.01–1.14)	1515 (284,773)	5.32 (5.19–5.46)
**Severe mental illness**		181,865	159 (141–177)	51	15	8	26	5135 (76,184)	2.44	67.42 (66.48–68.36)	650 (76,905)	8.44 (8.11–8.78)	50 (76,961)	0.64 (0.55–0.73)	155 (76,967)	2.01 (1.86–2.18)
**Transplant**	Kidney (with previous dialysis)	5745	179 (172–191)	35	18	11	37	395 (2745)	4.83	143.51 (136.46–150.93)	210 (2787)	76.08 (71.03–81.49)	50 (2800)	17.86 (15.5–20.57)	55 (2804)	18.9 (16.47–21.68)
	Kidney (without previous dialysis)	4515	179 (171–191)	36	19	11	35	280 (2160)	4.65	130.07 (122.54–138.06)	130 (2192)	58.4 (53.46–63.8)	25 (2202)	10.45 (8.48–12.87)	30 (2203)	14.52 (12.17–17.33)
	Other organ	6780	178 (170–188)	35	18	11	36	405 (3202)	3.91	127.12 (120.97–133.58)	165 (3247)	50.2 (46.42–54.29)	40 (3259)	11.66 (9.91–13.71)	55 (3262)	16.86 (14.73–19.29)
**Time since being fully vaccinated**	0–4 weeks	331,535	16 (8–23)	63	19	7	11	1100 (13,569)	5.91	81.14 (78.73–83.62)	170 (13,580)	12.37 (11.45–13.36)	REDACTED	REDACTED	80 (13,582)	5.74 (5.13–6.43)
	4–8 weeks	886,870	44 (37–51)	59	20	9	12	5135 (105,598)	7.09	48.65 (47.97–49.33)	335 (105,819)	3.15 (2.98–3.32)	20 (105,825)	0.18 (0.14–0.23)	125 (105,826)	1.17 (1.07–1.28)
	8–12 weeks	1,430,095	69 (62–76)	50	22	11	17	19,585 (270,294)	6.32	72.45 (71.94–72.97)	610 (271,783)	2.25 (2.16–2.34)	55 (271,801)	0.20 (0.18–0.23)	270 (271,804)	0.99 (0.93–1.05)
	12+ weeks	12,853,055	159 (135–184)	44	19	10	27	553,960 (5,523,038)	2.92	100.3 (100.16–100.43)	27,470 (5,602,353)	4.90 (4.87–4.93)	1910 (5,604,799)	0.34 (0.33–0.35)	5965 (5,604,967)	1.06 (1.05–1.08)
**Time between vaccinations**	6 weeks or less	499,185	185 (111–283)	39	18	11	31	17,760 (253,744)	2.43	69.99 (69.47–70.52)	1630 (256,550)	6.35 (6.20–6.51)	85 (256,777)	0.34 (0.03–0.38)	450 (256,788)	1.75 (1.67–1.83)
	6–12 weeks	13,565,570	150 (108–179)	46	19	10	25	519,470 (5,161,343)	3.20	100.65 (100.51–100.79)	23,120 (5,233,675)	4.42 (4.39–4.45)	1635 (5,235,630)	0.31 (0.03–0.32)	5005 (5,235,771)	0.96 (0.94–0.97)
	12 weeks or more	1,436,800	143 (100–170)	53	18	8	21	42,550 (497,411)	3.19	85.54 (85.13–85.96)	3830 (503,309)	7.61 (7.49–7.73)	270 (503,599)	0.53 (0.05–0.57)	980 (503,619)	1.95 (1.89–2.01)
**Prior COVID-19**	Between 1st and 2nd vaccination	191,320	65 (42–166)	48	19	9	24	1720 (49,491)	1.14	34.75 (33.93–35.6)	160 (49,786)	3.25 (3.01–3.52)	REDACTED	REDACTED	55 (49,815)	1.08 (0.95–1.24)
	Before 1st vaccination	926,960	138 (88–170)	42	19	10	29	8895 (326,947)	1.19	27.21 (26.92–27.5)	755 (328,374)	2.31 (2.22–2.39)	30 (328,454)	0.09 (0.08–0.11)	200 (328,458)	0.61 (0.57-0.66)

^a^All outcomes calculated as of 01st November 2021. ^b^All counts (of people and events) have been rounded to the nearest 5 and counts. ^c^Tests conducted is a count of (positive and negative) test results and may include multiple tests for an individual person

### COVID-19-related hospital admission

From within the fully vaccinated population, 28,580 had a COVID-19-related hospital admission (about 1 in 550 or 0.18%), with a median time to hospitalisation of 143 days (IQR 102–174). Of those who had a COVID-19-related hospital admission, 18,800 (66%) had a positive SARS-CoV-2 test in their records; 9170 (49%) occurred prior to an individual being hospitalised, 7030 (37%) occurred within the first 2 days of their hospital admission, 2365 (13%) occurred between 3 and 29 days after being hospitalised and 235 (1%) occurred 30 days or more after an individual had been hospitalised.

Rates of COVID-19-related hospital admissions increased with age: 1.09 (1.84–1.96) for 16–29 years old vs 12.75 (12.61–12.89) for those over 80 years, respectively. Rates were higher in those who were more deprived; most deprived IMD quintile versus least deprived IMD quintile was 7.32 (7.23–7.41) vs 3.55 (3.5–3.61), respectively. Comorbidities with the highest rates of COVID-19-related hospital admissions included kidney transplant, dialysis, and chronic kidney disease: 76.08 (95% CI 71.03–81.49), 70.73 (95% CI 63.34–78.99), and 49.49 (95% CI 45.33–54.02), respectively.

### COVID-19-related critical care admission

Of the 28,580 COVID-19-related hospital admission, 1980 needed to be admitted to critical care (about 1 in 1000 of fully vaccinated people or 1 in 14 of hospitalised patients), with a median time to critical care admission of 136 days (IQR 94-166). For every 1000 years of patient follow-up time, the corresponding incidence rate was ​0.33 (95% CI 0.32–0.34). Within the initial JCVI priority groups, COVID-19-related critical care admissions rates were highest in the CEV group (1.73, 95% CI 1.66–1.80) and lowest in health care workers 0.10 (95% CI 0.08–0.12).

Rates of COVID-19-related critical care admissions were higher in males than females and in; 0.45 (95% CI 0.44–0.46) vs 0.23 (95% CI 0.22–0.24), respectively. Rates were higher in those who were obese compared to not obese; BMI >30 (kg/m^2^) versus BMI <30 (kg/m^2^) was 0.68 (0.66–0.71) vs 0.22 (0.21-0.23), respectively. Comorbidities with the highest rates of COVID-19-related critical care admissions included dialysis, kidney transplant and chronic kidney disease: 19.74 (95% CI 16.03–24.32), 17.86 (95% CI 15.5–20.57) and 5.31 (95% CI 4.06–6.93), respectively.

### COVID-19-related death

Of those who were fully vaccinated, 6435 had a COVID-19-related death (about 1 in 2500 or 0.04%) with a median number of days to the death of 165 (IQR 129–188). While the majority (43%) of deaths occurred in the 80+ JCVI priority group, COVID-19-related death rates were three times as high amongst individuals living in care homes compared to those over 80 years living in private residences: 13.37 (95% CI 12.86–13.91) vs 4.64 (95% CI 4.56–4.73), respectively.

There were very few deaths in those under 30 years of age with rates of COVID-19-related death increasing with increased age: 0.02 (95% CI 0.02–0.03) for 16–29 years old vs 5.26 (95% CI 5.17–5.35) for those over 80 years, respectively. Comorbidities with the highest rates of COVID-19-related death included dialysis, transplant and chronic kidney disease: 25.71 (95% CI 21.42–30.86), 18.9 (95% CI 16.47–21.68), and 17.81 (95% CI 15.40–20.61), respectively.

## Conclusions

### Summary

This descriptive analysis in over 15 million people living in England shows that COVID-19 vaccine breakthrough is occurring; however, events are currently rare and mostly mild in nature. Nevertheless, some individuals are experiencing higher rates of serious illness and death due to COVID-19, such as those in care homes, with chronic kidney disease, on dialysis, with a transplant or who are immunosuppressed. It is important to note that this analysis is a simple descriptive piece of analysis; rates presented here are crude, and are subject to the limitations described below, making interpretation of potential differences in rates of outcomes, such as when stratified by time since vaccination, challenging. While it is possible to adjust rates (as demonstrated for patients with chronic kidney disease in Supplementary file 1) to help inform decisions around rollout of vaccine/booster programme for patients at high risk of adverse outcomes, it is important to stress that this study was not designed to estimate risk factors for COVID-19 vaccine breakthrough and thus cannot be used to answer why certain groups have higher rates of breakthrough cases than others or to estimate vaccine effectiveness.

### Strengths and weaknesses

This study used large-scale, routinely-collected primary care records, linked to coronavirus testing surveillance, hospital, and death registry data. This allowed us to describe a substantial proportion of the English population in rich longitudinal detail and to detect variations in COVID-19 vaccine breakthrough cases, as early as possible.

We acknowledge several important limitations of these findings. First, even though the base population consisted of over 15 million fully vaccinated individuals the numbers of COVID-19 vaccine breakthrough cases were relatively small, especially for hospitalisations and deaths. This made comparisons between outcomes, specifically at selected clinical and demographic levels difficult, meaning rates could be imprecisely estimated. Second, due to the targeted rollout of the COVID-19 vaccination programme in England, this cohort represents mostly older and vulnerable populations. In addition, follow-up time is systematically different amongst individuals included in this study and no adjustment for this has been made. Third, asymptomatic testing patterns vary between individuals. Apart from health and care workers, asymptomatic individuals without any underlying health issues or comorbidities are less likely to get tested than those with underlying health issues or comorbidities (i.e., haemodialysis patients) who will undergo asymptomatic testing regularly. Most lateral flow tests conducted at home are not included in our data. The number of reported positive SARS-CoV-2 tests is therefore likely to be an undercount of SARS-CoV-2 amongst fully vaccinated individuals without any underlying health issues or comorbidities, which may have led to underestimation of the corresponding rates. Fourth, while individuals were excluded if they tested positive for SARS-CoV-2 or had been hospitalised or died due to COVID-19 within the two weeks after their second dose, we acknowledge that there is still the potential that some patients will have been included whose outcome was related to a previous infection pre second vaccination. In particular, this may affect some of the highest risk groups such as dialysis patients and those in care homes where there is a high amount of repeated screening in and outcomes stratified by time since vaccination. As a result, there may be a potential increase in the associated rates; as indicated by the high positive SARS-CoV-2 test incidence rate in those 0–4 weeks following full vaccination. Fifth, characteristics linked to COVID-19 vaccine breakthrough in fully vaccinated individuals may be reflective of higher infection rates regardless of vaccination in some groups, not because of vaccination (i.e. due to higher exposure due to behavioural differences) [18]. Finally, this study includes data from December 2020 until November 2021, during which time two different variants of SARS-COV-2 were dominating in England at different times; the Alpha variant (B.1.1.7) between January 2021 and May 2021, and the Delta variant (B.1.617.2) after June 2021 [19]. These have since been supplanted by other variants, in particular the Omicron variant. This is a descriptive study and does not aim to quantify the contribution of vaccine breakthrough due to the change in dominant variant from Alpha to Delta or the extent of waning in the months immediately following vaccination. Further studies are needed to assess vaccine waning and rates of breakthrough COVID-19 between different variants and in newer, more prevalent variants, such as Omicron (B.1.1.529).

### Findings in context

There is strong evidence that the UK’s COVID-19 vaccination programme has reduced infection and severe outcomes in vaccinated individuals [4, 5]. However, breakthrough infections after vaccination against SARS-CoV-2 are increasingly reported [7] and there are concerns that the effectiveness of the vaccines may reduce over time, due to the vaccines being less effective against the delta variant, and/or the waning of the immune response [8]. While we did find that breakthrough infections were more frequent in the earlier-vaccinated individuals, potentially due to waning vaccine efficacy or impaired immune response, further studies are needed to gain a clearer picture of the long-term effectiveness of COVID-19 vaccines. In addition, with only a handful of studies investigating risk factors for post-vaccination infection [13, 16, 17], very little is known about how breakthrough COVID-19 varies between key clinical and demographic groups. Our findings on COVID-19 vaccine breakthrough are consistent with patterns observed worldwide; COVID-19 vaccine breakthrough cases are rare and mild. However, there are potentially several groups who are at higher risk of COVID-19 vaccine breakthrough including those in care-homes, with chronic kidney disease, on dialysis, with transplants or who are immunocompromised.

## Conclusion

As of 1st November 2021, the majority of COVID-19 vaccine breakthrough cases in England were mild with relatively smaller numbers of fully vaccinated individuals being hospitalised or dying as a result of COVID-19. While these numbers are in line with expectations, and while follow-up time is systematically different amongst fully vaccinated individuals and variation in vaccination coverage between groups and regions will have many complex drivers, some potential differences in rates of breakthrough cases were identified in several clinical groups. The emergence of the Omicron variant of COVID-19 coupled with the number of positive SARS-CoV-2 tests still occurring is concerning and as numbers of fully vaccinated (and boosted) individuals increases and as follow-up time lengthens, so too will the number of COVID-19 breakthrough cases. Additional analyses are therefore, to assess vaccine waning and rates of breakthrough COVID-19 between different variants, and to enable identification of individuals at higher risk, who would require continued strict precautions, and additional vaccination.

## Supplementary Information

**Additional file 1. **This additional piece of analysis uses patients with chronic kidney disease to show how rates can be adjusted to demonstrate the public health burden and to help inform decisions around rollout of vaccine/booster programme for patients at high risk of adverse outcomes, **Table S1.** Number of fully vaccinated (2 doses + 2 weeks) patients with chronic kidney disease by stage, in OpenSAFELY-TPP, and associated crude and adjusted rates of positive SARS-CoV-2 swab test, COVID-19 related hospital admissions, COVID-19 related critical care admissions and COVID-19 related death, broken down by CKD stage.

## Data Availability

Access to the underlying identifiable and potentially re-identifiable pseudonymised electronic health record data is tightly governed by various legislative and regulatory frameworks, and restricted by best practice. The data in OpenSAFELY is drawn from General Practice data across England where TPP is the Data Processor. TPP developers (CB, RC, JP, FH, and SH) initiate an automated process to create pseudonymised records in the core OpenSAFELY database, which are copies of key structured data tables in the identifiable records. These are linked onto key external data resources that have also been pseudonymised via SHA-512 one-way hashing of NHS numbers using a shared salt. DataLab developers and PIs (BG, LS, CEM, SB, AJW, WH, DE, PI, and CTR) holding contracts with NHS England have access to the OpenSAFELY pseudonymised data tables as needed to develop the OpenSAFELY tools. These tools in turn enable researchers with OpenSAFELY Data Access Agreements to write and execute code for data management and data analysis without direct access to the underlying raw pseudonymised patient data, and to review the outputs of this code. All code for the full data management pipeline—from raw data to completed results for this analysis—and for the OpenSAFELY platform as a whole is available for review at github.com/OpenSAFELY.

## Abbreviations

BMI: Body mass index
JCVI: Joint Committee Vaccination and Immunisation
IMD: Index of multiple deprivation
UK: United Kingdom
