# Supplementary material for: Describing the population experiencing COVID-19 vaccine breakthrough following second vaccination in England: a cohort study from OpenSAFELY
Source: BMC Med. 2022 Jul 5;20:243. doi: 10.1186/s12916-022-02422-0 (PMC9255436; doi:10.1186/s12916-022-02422-0)
Supplement: Supplementary file 1 — Additional file 1. This additional piece of analysis uses patients with chronic kidney disease to show how rates can be adjusted to demonstrate the public health burden and to help inform decisions around rollout of vaccine/booster programme for patients at high risk of adverse outcomes, Table S1. Number of fully vaccinated (2 doses + 2 weeks) patients with chronic kidney disease by stage, in OpenSAFELY-TPP, and associated crude and adjusted rates of positive SARS-CoV-2 swab test, COVID-19 related hospital admissions, COVID-19 related critical care admissions and COVID-19 related death, broken down by CKD stage. [file 12916_2022_2422_MOESM1_ESM.pdf]

Supplementary material

Chronic kidney disease

To demonstrate the public health burden (rather than likely causal mechanisms), within patients with chronic kidney disease (CKD), Table S1 shows both crude and adjusted rates of positive SARS-CoV-2 swab test, COVID-19 related hospital admissions, COVID-19 related critical care admissions and COVID-19 related death, broken down by CKD stage. Incidence rates for each outcome were estimated by dividing the count by person-years, with 95% confidence intervals. Crude and adjusted rates were also calculated using cox regression and show that even after adjustment, rates remain very high. These results highlight that fully vaccinated individuals with kidney disease are at far higher risk of serious outcomes when infected with COVID-19 than individuals without kidney disease.

Table S1: *Number of fully vaccinated (2 doses + 2 weeks) patients with chronic kidney disease by stage, in OpenSAFELY-TPP, and associated crude and adjusted rates of positive SARS-CoV-2 swab test, COVID-19 related hospital admissions, COVID-19 related critical care admissions and COVID-19 related death, broken down by CKD stage.*

**Population/Outcome**			**Kidney disease**				
			**No CKD**	**Stage 3a**	**Stage 3b**	**Stage 4**	**Stage 5**
**Fully vaccinated**	**Count***		14684665	529000	206450	51210	5505
	**Follow-up time, medium days (IQR)**		147 (103-177)	187 (173-201)	192 (178-205)	191 (174-204)	184 (164-200)
	**Tests conducted (%)**	**0**	46	53	52	47	29
		**1**	19	16	14	13	12
		**2**	10	8	7	8	7
		**3+**	25	23	27	33	52

Population/Outcome		Kidney disease				
		No CKD	Stage 3a	Stage 3b	Stage 4	Stage 5
Positive SARS-CoV-2 test	Positivity rate, %	3.21	2.18	2.16	2.19	1.75
	Events* (PYs)	560175 (5496274)	12170 (268808)	4450 (107113)	1280 (25907)	215 (2607)
	Incidence rate per 1000 person-years (95% CI)	101.92 (101.78-102.06)	45.27 (44.87-45.69)	41.56 (40.95-42.19)	49.41 (48.05-50.81)	82.47 (77.04-88.29)
	Hazard ratio	1.00	0.4 (0.39-0.41)	0.37 (0.36-0.38)	0.44 (0.42-0.46)	0.75 (0.65-0.85)
	Age-adjusted hazard ratio	1.00	0.78 (0.77-0.8)	0.8 (0.78-0.82)	0.94 (0.89-1)	1.28 (1.12-1.47)
COVID-19 related hospital admission	Events* (PYs)	21480 (5575237)	3370 (270184)	2105 (107527)	780 (26000)	130 (2627)
	Incidence rate per 1000 person-years (95% CI)	3.85 (3.83-3.88)	12.48 (12.27-12.7)	19.59 (19.16-20.02)	29.92 (28.87-31.01)	49.49 (45.33-54.02)
	Hazard ratio	1.00	2.27 (2.19-2.36)	3.38 (3.23-3.53)	5.25 (4.89-5.64)	9.34 (7.86-11.1)
	Age-adjusted hazard ratio	1.00	1.76 (1.7-1.83)	2.5 (2.38-2.62)	3.87 (3.59-4.16)	7.49 (6.3-8.91)
COVID-19 related critical care admission	Events* (PYs)	1505 (5577078)	190 (270500)	95 (107716)	45 (26064)	15 (2638)
	Incidence rate per 1000 person-years (95% CI)	0.27 (0.26-0.28)	0.7 (0.65-0.75)	0.89 (0.8-0.99)	1.8 (1.56-2.09)	5.31 (4.06-6.93)
	Hazard ratio	1.00	1.93 (1.66-2.25)	2.39 (1.94-2.94)	4.94 (3.69-6.61)	15.41 (9.1-26.1)
	Age-adjusted hazard ratio	1.00	1.81 (1.54-2.13)	2.22 (1.79-2.76)	4.58 (3.4-6.17)	14.6 (8.61-24.77)

Population/Outcome		Kidney disease				
		No CKD	Stage 3a	Stage 3b	Stage 4	Stage 5
COVID-19 related death	Events* (PYs)	3985 (5577213)	1080 (270514)	795 (107722)	335 (26067)	45 (2638)
	Incidence rate per 1000 person-years (95% CI)	0.71 (0.7-0.73)	4 (3.88-4.12)	7.39 (7.13-7.66)	12.85 (12.17-13.57)	17.81 (15.4-20.61)
	Hazard ratio	1.00	3.37 (3.15-3.61)	5.69 (5.27-6.14)	10.11 (9.03-11.31)	15.58 (11.68-20.78)
	Age-adjusted hazard ratio	1.00	1.59 (1.48-1.71)	2.27 (2.09-2.46)	3.87 (3.45-4.34)	7.81 (5.85-10.42)
